# Professional Reputation

**Published:** 1901-10-19

**Authors:** 


### The Hospital

A JOURNAL OF

The Medical Sciences and Hospital Administration.

VOL. XXXI.—NO. 786.] EDITED BY SIR HENRY BURDETT, K.C.B., AND SOLOMON C. SMITH, M.D., M.R.C.P. [OCTOBER 19, 1901.

### PROFESSIONAL REPUTATION.

THE recently published memoirs of the late Sir James Paget, founded upon a brief chronicle of the events of his life prepared by himself in the period between his seventieth and seventy-fifth years, and containing, here and there among the narrative, his reflections and conclusions with regard to many of the problems which had been forced upon his attention by the incidents of his professional career, will furnish many occasions for self-questioning to the majority of medical readers. The circumstances and qualities which open the door to successful practice, the various "ways out" of such a practice when it has been obtained, the methods of self-examination rendered necessary by the gradual advance of age, and the possible varieties of "professional reputation," are all treated with that remarkable blending of simplicity with shrewdness, that keen perception of the limitations imposed by circumstances upon the attainment of the abstractedly desirable, which formed such prominent features of Paget's character; and all that he has to say regarding them may be studied with much advantage, not only by practitioners but also by the public, whose estimate of the capacities and the trustworthiness of their medical attendants, or of those who seek their suffrages in that capacity, has not always been guided by sound knowledge in times past, and seems likely to be still less so guided in the future. Whenever a Paget again rises amongst us, he will take his place in the front rank by an inevitable process of natural selection; but, in the meanwhile, and in the presence only of more ordinary types of humanity, it would seem as if the road to professional prosperity might as often be devious as direct. It is much to the interest of the public, it is still more to the interest of the profession, that this reproach should be removed from modern civilisation.

After describing the large extent to which his success as a "rising man" was due to the good opinion of his fellow practitioners, and after saying that this should be the proper road to their goal for any who have either reasonable ambition or reasonable grounds for thinking themselves able to gain a leading place, more especially for those whose work, as in the case of the holders of hospital appointments, must be done in the sight of competent observers who criticise it and talk about it every day, the memoir proceeds to describe the contrast between the reputation which may thus be gained, and that kind which is due to the opinion of patients, or of "whatever may be called the public." The contrast between the two, except in so far as this public opinion may depend on what is learned from the profession, is declared to be "nearly complete." The one is due to the opinions of those of whom the majority are fit to judge; the other, very often, to the mere talk of those of whom the majority are quite unable to form a reasonable opinion, and among whom the most positive, the most talkative, and the most influential are generally the most ignorant. Sir James wrote that he remembered these things and noted them, so as to have place for saying that, as reputation among the members of the profession may rightly be sought as a great motive to self-improvement, so reputation among the public alone can scarcely be sought, directly and on purpose, without great risk of damage. Such reputation, as measured only or chiefly by money, may be obtained by the most ignorant through self-assertion, self-advertisement, or mere impudence.

It is impossible to read these weighty words without carrying back the mind to the consideration of a certain proportion of the so-called "medical literature" under which the tables of reviewers have of late years been compelled to groan, and which has found its way quite as much to the editors of the non-medical as to those of the medical press. Nothing does quite so much harm to the profession as the successful career of a young gentleman possessed of sufficient "cleverness"—to use the most detestable of all words—to obtain a qualification sufficiently high-sounding for his purposes, and who then, without due check from any sense of duty or of responsibility, begins to sound his brazen trumpet in relation to diet, or to "open-air treatment," or to obesity, or to muscular exercises, or to any other subject which has for the moment obtained access to the columns of the newspapers, and which seems capable of being "worked" in a manner financially satisfactory. While men of science are engaged in researches which may some day throw useful light upon the processes of nutrition, and may enable genuine students of physiology and of organic chemistry really to exercise some control over those processes and over the methods and results of their perversion, the adventurers in question bring out their little books, saying, in effect, that the sufferers from the troubles described in them will find their only veritable Bethesda at 37 Queer Street, Impostor Square.

Was any little book on the "popular" medical question of the day ever published which had for its object the diffusion, either among the profession or among the public, of genuine knowledge with regard to the particular matter at issue, or which was anything else than an excuse for advertisements of the name of some writer who, in reality, knew no more about the subject of his treatise than was known to every man in the profession, and to many beyond it? We think not, and we think it high time that the heads of the medical corporations, who have large powers under their bye laws, and the senior members of hospital staffs, should take some steps in restraint of publications which, in the long run, do infinite harm to the medical profession as a whole. We will not say that the booklet writers are impostors; but, as a rule, they are people who lead their readers to entertain expectations which are not likely to be ealised. When such expectations are disappointed, the blame is seldom cast upon the right shoulders, but is, as often as not, attributed to the "uncertainties of medical science." Poor medical science, to be rendered responsible for all that is done in her name, often by those who are scarcely acquainted with her rudiments.

